# Summer Drinks

**Published:** 1873-05

**Authors:** 


### SUMMER DRINKS.

The most healthful drink is running water, at the temperature of the air at the time and place. Running water purifies itself. The running water of a spring is purer fifty feet from the fountain than at the fountain head, because exposure to the air causes the oxidation or chemical decomposition of any impure and more solid matters which bubble up from the depths below. Many cases are given where the drinking of apparently pure spring or well water has caused severe sickness or death in whole families, because they received the drainings of privies and barn-yards and house-drains.

A pint of molasses stirred in a gallon of water makes a healthy, cooling, opening drink for summer, and is very agreeable; is good for harvesters, and so is buttermilk.

It is a great mistake on the part of families to get into the habit of using root beer, ginger tea, cider and lemonade, as a daily drink, in summer. They increase thirst and otherwise derange the system.

Buttermilk is also a delightful, safe and nourishing drink for all, especially laborers and men in the harvest field.

Acid drinks in summer cool the system, correct biliousness, and keep down fevers. Those made with lemon, sugar and water are better than if vinegar is used.

If ice-water is used it should be between meals, with an interval of a few seconds after each swallow or two. This is always safe, and too much cannot be drank without forcing. Half a glass drank thus, will satisfy more than a pint if drank continuously, and without the uncomfortable sensation of fulness accompanying large potations. It is more healthful and far better to take a cup of hot drink at meals than a glass of ice water; the latter has often given dangerous, and even fatal chills. No one is injured by a cup of hot tea. If a person sits down to a meal on a hot day—weak and tired, and hungry and thirsty—a glass of ice-water will have a delicious taste; but it endangers an ugly chill, while a cup of tea is absolutely reviving and invigorating, raising the spirits, and preparing the stomach for the reception of a hearty meal.

Let it be remembered, that the sensation of thirst is as completely satisfied with a glass of water as with a glass of soda, beer, or any spirit mixture, with the advantage that it costs nothing. Any one may try this for himself, when, on a warm day, he feels that a glass of soda water or iced toddy would be perfectly delicious. Let him take, instead, a glass of pure, cool water, then he would not take the liquor if he could get it. He has no desire for it.

